# A SIRT6 Inhibitor, Marine-Derived Pyrrole-Pyridinimidazole Derivative 8a, Suppresses Angiogenesis

**DOI:** 10.3390/md21100517

**Published:** 2023-09-28

**Authors:** Nannan Song, Yanfei Tang, Yangui Wang, Xian Guan, Wengong Yu, Tao Jiang, Ling Lu, Yuchao Gu

**Affiliations:** 1 Key Laboratory of Marine Drugs, Ministry of Education, School of Medicine and Pharmacy, Ocean University of China, Qingdao 266003, Chinajiangtao@ouc.edu.cn (T.J.); 2 Laboratory for Marine Drugs and Bioproducts of Laoshan Laboratory, Qingdao 266237, China; 3 College of Marine Science and Biological Engineering, Qingdao University of Science and Technology, Qingdao 266042, China

**Keywords:** SIRT6 inhibitor, anti-angiogenesis, tube formation, anticancer

## Abstract

Angiogenesis refers to the process of growing new blood vessels from pre-existing capillaries or post-capillary veins. This process plays a critical role in promoting tumorigenesis and metastasis. As a result, developing antiangiogenic agents has become an attractive strategy for tumor treatment. Sirtuin6 (SIRT6), a member of nicotinamide adenine (NAD^+^)-dependent histone deacetylases, regulates various biological processes, including metabolism, oxidative stress, angiogenesis, and DNA damage and repair. Some SIRT6 inhibitors have been identified, but the effects of SIRT6 inhibitors on anti-angiogenesis have not been reported. We have identified a pyrrole-pyridinimidazole derivative **8a** as a highly effective inhibitor of SIRT6 and clarified its anti-pancreatic-cancer roles. This study investigated the antiangiogenic roles of **8a**. We found that **8a** was able to inhibit the migration and tube formation of HUVECs and downregulate the expression of angiogenesis-related proteins, including VEGF, HIF-1α, p-VEGFR2, and N-cadherin, and suppress the activation of AKT and ERK pathways. Additionally, **8a** significantly blocked angiogenesis in intersegmental vessels in zebrafish embryos. Notably, in a pancreatic cancer xenograft mouse model, **8a** down-regulated the expression of CD31, a marker protein of angiogenesis. These findings suggest that **8a** could be a promising antiangiogenic and cancer therapeutic agent.

## 1. Introduction

Angiogenesis, which refers to the development of new blood vessels from existing capillaries or post-capillary veins, plays a critical role in tumor growth and metastasis [1,2]. As tumors grow, they consume nutrition and oxygen in the microenvironment, prompting the rapid development of new blood vessel networks to supply more nutrients and oxygen to the tumor, thereby promoting tumor growth and metastasis [3,4]. Therefore, the development of antiangiogenic inhibitors is of great value for the treatment of tumors, and antiangiogenic therapy has received widespread attention in the treatment strategy of solid tumors over the past few decades.

Sirtuin6 (SIRT6) is a member of the sirtuin family of nicotinamide adenine (NAD^+^)-dependent histone deacetylases. SIRT6 has been proven to be involved in regulating diverse biological processes, including metabolism, oxidative stress, DNA damage, and repair, and plays a significant role in aging, cancer, inflammation, diabetes, and other diseases [5,6,7,8]. Several studies have reported that SIRT6 is involved in the regulation of angiogenesis. For instance, SIRT6 promotes angiogenesis by inhibiting the ubiquitination degradation of HIF-1α and promoting its expression in carotid artery plaque [9]. SIRT6 overexpression promotes angiogenesis and reduces cerebral ischemia and reperfusion (I/R)-induced injury through transcriptional inhibition of TXNIP [10]. In addition, recent reports have found that MDL-800, a highly efficient SIRT6 activator, can inhibit the NF-κB signaling pathway by activating SIRT6, promoting angiogenesis, and wound healing [11]. More importantly, in pancreatic cancer, SIRT6 could promote the expression of inflammatory factor IL-8, which could promote local inflammation and further promote angiogenesis, playing a key role in the occurrence and metastasis of pancreatic cancer [12,13,14]. These studies suggest that SIRT6 is a new anti-angiogenesis target, and inhibitors of SIRT6 may have a highly effective antiangiogenic effect.

**Figure 1:**
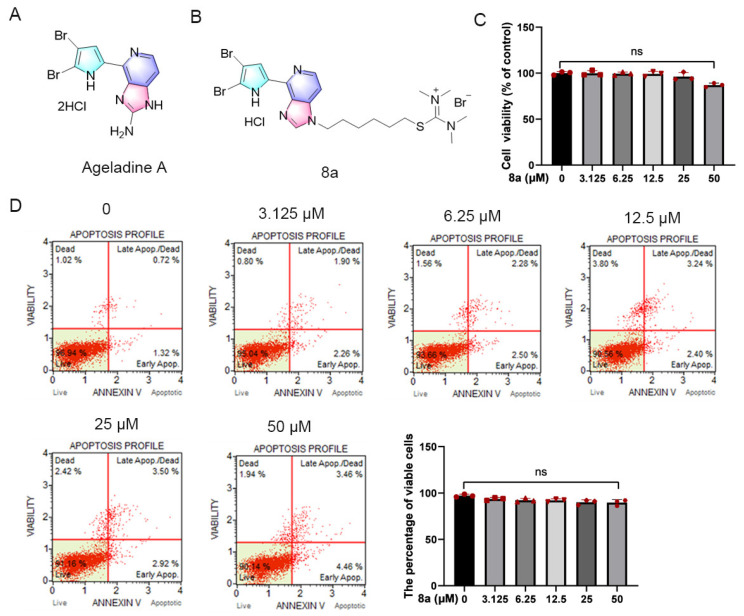
Compound **8a** did not induce toxicity in HUVECs. (**A**) Chemical structure of Ageladine A. (**B**) Chemical structure of **8a**. (**C**) HUVEC cells were treated with **8a** of 0, 3.125, 6.25, 12.5, 25, and 50 μM for 24 h; cell viability was determined by CCK8 assay. (**D**) The effect of **8a** on apoptosis was detected by flow cytometry. Data were presented as mean ± SD of three independent experiments.

Marine environments offer unique ecological conditions, making marine natural products a rich source of new bioactive agents [15,16,17,18]. Ageladine A (Figure 1A), a fluorescent pyrrol-2-aminoimidazole alkaloid extracted from the marine sponge *Agelas nakamurai* by Fusetani et al. has demonstrated significant activity as an inhibitor of matrix metalloproteinases (MMPs) such as MMP-1, 2, 8, 9, 12, and 13 at micromolar levels. These MMPs play crucial roles in apoptosis, metastasis, and angiogenesis of tumor cells [19,20]. In a prior study, we observed that the derivative of Ageladine A, **8a** (as shown in Figure 1B), displayed inhibitory effects on SIRT6 both in vivo and in vitro, while not affecting MMPs. Additionally, the derivative showed potential as an anti-pancreatic-cancer agent [21].

The objective of this study was to investigate the antiangiogenic roles of SIRT6 inhibitor **8a** both in vivo and in vitro. Our findings demonstrated that **8a** suppressed the migration and angiogenesis of HUVECs without inducing apoptosis in these cells. The observed effects were linked to the downregulation of key angiogenic molecules, such as N-cadherin, VEGF, and HIF-1α. Moreover, we determined that **8a** inhibited intersegmental vessel formation in a zebrafish model. Most notably, our study revealed that **8a** significantly inhibited the expression of the angiogenic marker protein CD31 and suppressed angiogenesis in a pancreatic cancer xenograft model. Our results strongly suggest that SIRT6 inhibitors possess great potential as novel antiangiogenic agents.

## 2. Results

### 2.1. Compound **8a** Has No Obvious Toxicity in HUVECs

Initially, we assessed the potential impact of **8a** on HUVEC cell viability through the CCK8 assay (Figure 1C). HUVEC cells were treated with varying concentrations of **8a** (0, 3.125, 6.25, 12.5, 25, and 50 μM) for 24 h, and we observed that compound **8a** did not significantly affect the viability of HUVEC cells. Only at a concentration of 50 μM did we observe a slight reduction in HUVEC cell viability. Additionally, we evaluated the effect of compound **8a** on apoptosis via flow cytometry (Figure 1D). After HUVEC cells were treated with varying concentrations of **8a** (0, 3.125, 6.25, 12.5, 25, and 50 μM) for 24 h and stained with Annexin V and PI, we observed over 90% of viable cells in all samples, with no significant differences between **8a**-treated and untreated cells. Our findings suggested that **8a** did not exhibit any toxic effects on HUVEC cells at the selected concentration. As a result, the concentration of **8a** we used in subsequent experiments was below 25 μM and excluded any potential cytotoxicity of the compound. 

### 2.2. Compound **8a** Inhibits Migration of HUVECs

**Figure 2:**
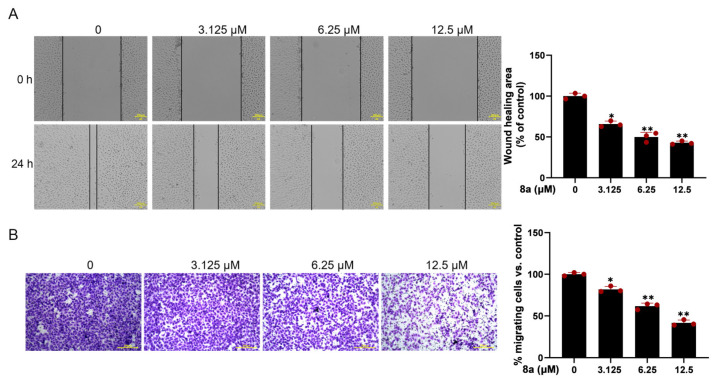
Compound **8a** inhibits the migration ability of HUVECs. (**A**) Representative images of wound healing assay of HUVECs at 0 and 24 h post-treatment with compound **8a** (magnification: ×200). The wound-healing area was determined using Image J software v1.53. Scale bar = 200 μm. (**B**) Transwell assay to detect the migration of HUVECs. Representative images of HUVECS traveling through membrane after treatment with different concentrations of **8a** were taken by a microscope. Scale bar = 200 μm. Data are presented as mean ± SD of three independent experiments. * *p* < 0.05, ** *p* < 0.01 versus control.

Since vascular endothelial cell migration is a crucial step in angiogenesis, we evaluated the effect of **8a** on HUVEC cell migration using the scratch-wound assay. The results (Figure 2A) demonstrated that the migration of HUVECs was significantly reduced after 24 h treatment with 0, 3.125, 6.25, and 12.5 μM **8a**, with a wound healing percentage of 65.8, 51.6, and 43.2%. To further confirm the effect of **8a** on HUVEC cell migration, we conducted the transwell assay. The results (Figure 2B) showed that the number of migrated cells was significantly reduced after treatment with **8a**, providing further evidence that **8a** could inhibit the migration of HUVEC cells.

### 2.3. Compound **8a** Inhibits Tube Formation Abilities of HUVECs

**Figure 3:**
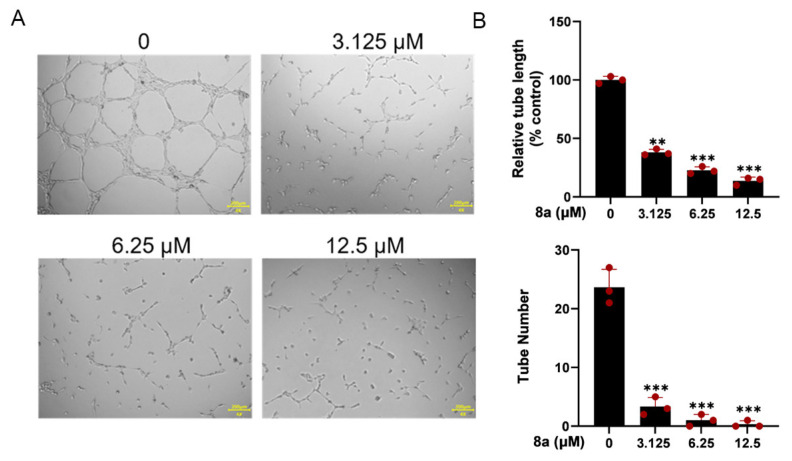
Compound **8a** inhibits the tube formation ability of HUVECs. (**A**) Tube formation ability of HUVECs treated with different concentrations of compound **8a** (0, 3.125, 6.25, and 12.5 μM) for 6 h. Scale bar = 200 μm. (**B**) Quantification of tube length and number. Data are presented as mean ± SD of three independent experiments. ** *p* < 0.01, *** *p* < 0.001 versus control.

The ability of HUVEC cells to form capillary-like structures within 6 h on a Matrigel-coated culture plate makes them a suitable model for studying angiogenesis in vitro [22]. Therefore, we used HUVEC cells to investigate the antiangiogenic effects of **8a** in vitro. As illustrated in Figure 3, our findings revealed that **8a** significantly reduced both the number and length of tubes formed by HUVECs, indicating that it could significantly inhibit angiogenesis. 

### 2.4. Compound **8a** Decreases the Expression of VEGF, p-VEGFR2, N-Cadherin, HIF-1α, p-AKT, and p-ERK in HUVECs

**Figure 4:**
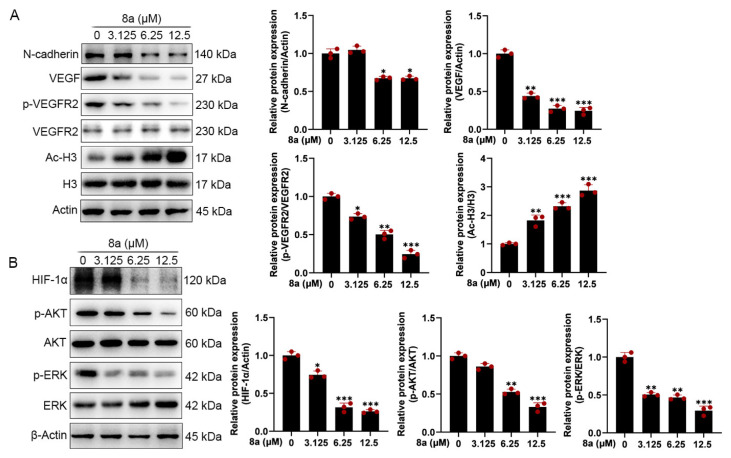
Compound **8a** decreases the expression of VEGF, p-VEGFR2, HIF-1α, and N-cadherin and the activation of AKT and ERK pathways which were associated with angiogenesis in HUVECs. (**A**) Western blot assay was used to detect the expression of VEGF, p-VEGFR2, VEGFR2, N-cadherin, H3, and Ac-H3 proteins after **8a** treatment for 24 h. (**B**) Western blot assay was used to detect the expression of HIF-1α, p-AKT, AKT, p-ERK, and ERK proteins after **8a** treatment for 24 h. Data are presented as mean ± SD of three independent experiments. * *p* < 0.05, ** *p* < 0.01, *** *p* < 0.001 versus control.

To further explore the effect of **8a** on the molecular mechanism related to angiogenesis, we detected the expression of angiogenesis-related proteins by Western blot. As shown in Figure 4A, we found that **8a** treatment of HUVEC cells significantly upregulated the acetylation level of SIRT6 downstream target protein H3, indicating that **8a** could inhibit the activity of SIRT6 in HUVEC cells. Vascular endothelial growth factor (VEGF) plays an important role in angiogenesis, causing the proliferation and migration of vascular endothelial cells and increasing vascular permeability by phosphorylating VEGF receptor-2 (VEGFR-2) [23,24,25,26]. We found that **8a** could significantly downregulate the expression of VEGF and p-VEGFR2 in a concentration-dependent manner. N-cadherin is a transmembrane glycoprotein that mediates vascular formation and structural integrity and is involved in the regulation of cell invasion and metastasis [27,28,29,30]. We examined the effect of **8a** on the expression level of N-cadherin by Western blot assay and found that **8a** could significantly inhibit the expression of N-cadherin in a concentration-dependent manner.

HIF-1α is an important transcription factor that can be involved in promoting angiogenesis by regulating VEGF [31]. SIRT6 promotes angiogenesis by inhibiting the ubiquitination degradation of HIF-1α and promoting its expression [9]. Therefore, we evaluated the effect of **8a** on HIF-1α and found that **8a** significantly inhibited the expression of HIF 1α (Figure 4B). Activation of pathways can also contribute to promoted angiogenesis by regulating the expression of factors such as VEGF [32,33]. Therefore, we also examined the effect of **8a** on the phosphorylation of AKT and ERK by Western blot, and we found that **8a** could downregulate the levels of p-AKT and p-ERK (Figure 4B). These results suggest that compound **8a** inhibits angiogenesis in HUVEC cells by suppressing the activation of AKT and ERK pathways and the expression of VEGF, N-cadherin, and HIF-1α.

### 2.5. Compound **8a** Inhibits Intersegmental Vessel Formation in Zebrafish Model

**Figure 5:**
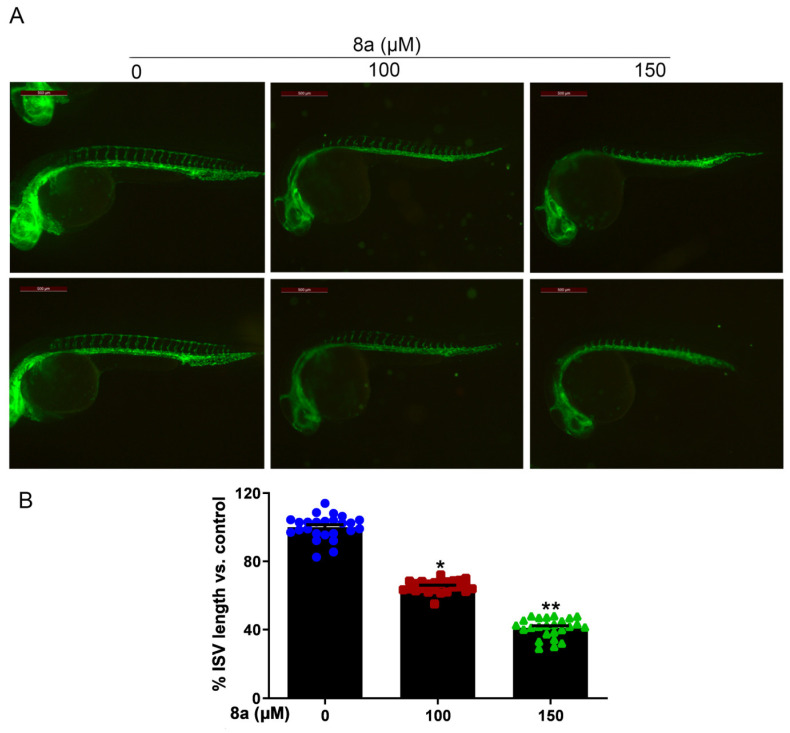
Compound **8a** blocks angiogenesis of intersegmental vessels in zebrafish embryos (N = 25, each group). (**A**) Representative vessel images of *Tg (flk1: EGFP)* zebrafish embryos treated with different concentrations of **8a**. Scale bar = 500 μm. (**B**) Quantitative analysis of intersegmental vessel formation induced by compound **8a**. Data are presented as mean ± SD of three independent experiments. * *p* < 0.05, ** *p* < 0.01 versus control.

Zebrafish is an effective model organism for studying vascular development, owing to its highly characteristic vascular pattern and rapid vascular development [34,35]. Compared to other animal models, zebrafish offers several advantages [36,37]. The zebrafish genome has more than 95% similarity to human genes in function, particularly those related to angiogenesis. Angiogenesis in zebrafish starts at 12 hours post fertilization (hpf) and, by 20 hpf, its dorsal aorta (DA) and cardinal vein (CV) are already formed, with new vessels gradually extending towards the trunk and tail and intersegmental vessels (ISVs) sprouting from the aorta. By 48 hpf, the ISVs of the trunk and tail are largely formed and blood circulation is established [38,39,40]. Furthermore, various artificially mutated strains of zebrafish, such as the *Tg (flk1: EGFP)* zebrafish, allow for direct observation of vascular development dynamics via fluorescence microscopy [41,42,43]. Given the multiple advantages of zebrafish, this model organism has been successfully used to evaluate or screen different tissues for antiangiogenic agents on the vasculature [44,45]. Therefore, we employed *Tg (flk1: EGFP)* zebrafish to evaluate the effect of compound **8a** on angiogenesis in vivo. We treated *Tg (flk1: EGFP)* zebrafish with different concentrations of **8a** for 28 h and examined the formation of ISVs in zebrafish embryos. Our results (Figure 5) showed that **8a** inhibited ISV formation in zebrafish embryos by 34.57% and 59.87% at 100 and 150 μM, respectively. This illustrates that compound **8a** can inhibit angiogenesis in zebrafish and has no significant effect on mature vessels formed before drug treatment.

### 2.6. Compound **8a** Suppresses Tumor Angiogenesis in Pancreatic Cancer Xenograft Model

**Figure 6:**
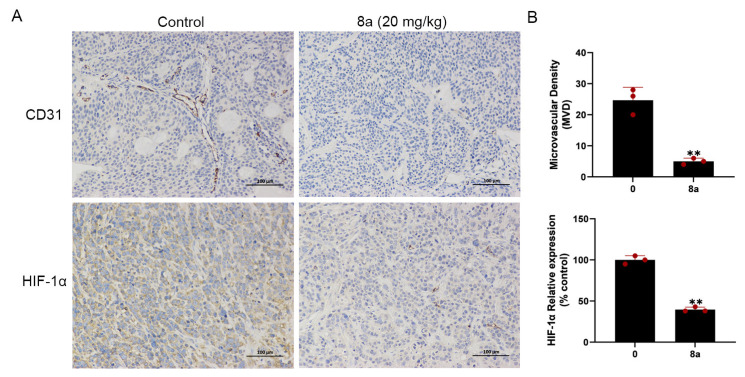
Compound **8a** suppressed tumor angiogenesis in pancreatic cancer xenograft model (N = 6, each group). (**A**) Representative tumor tissue sections from BXPC-3 xenograft tumors with CD31 and HIF-1α staining after treatment with compound **8a**. Scale bar = 100 μm. (**B**) Quantification analysis of newly formed vessels corresponding to immunohistochemical staining of CD31 and HIF-1α. Data are presented as mean ± SD. ** *p* < 0.01 versus control.

We conducted additional experiments to assess the impact of compound **8a** on angiogenesis in pancreatic cancer. CD31 is a marker protein that is commonly used to detect neovascularization and is localized at the border between vascular endothelial cells and newly formed blood vessels and lymphatic vessels [46,47]. HIF-1α can be involved in promoting angiogenesis by regulating VEGF [31]. In our previous research, we found that **8a** inhibited the growth of pancreatic tumors in the pancreatic cancer xenograft model and, on this basis, we evaluated the angiogenesis of pancreatic cancer by detecting the expression level of CD31 and HIF-1α using immunohistochemical staining. Our results (Figure 6) showed a reduction in the level of CD31 and HIF-1α protein expression after treatment with **8a**, indicating that compound **8a** inhibited angiogenesis in pancreatic cancer.

## 3. Discussion

Numerous studies have shown that the rapid growth and metastasis of tumors require blood vessels to provide oxygen and nutrients, without which they can only remain dormant [1,48,49]. Angiogenesis plays an important role in promoting tumorigenesis and metastasis; therefore, the development of antiangiogenic agents is an attractive strategy for tumor treatment. Previous research has demonstrated that SIRT6 promotes angiogenesis, suggesting that SIRT6 is an antiangiogenic target. We previously found that Ageladine A derivative **8a**, a novel and highly effective inhibitor of SIRT6, can inhibit the activity of SIRT6 and has anti-pancreatic-cancer activity in vivo and in vitro. However, its anti-angiogenesis activity has not been explored. In this study, we demonstrated for the first time that **8a**, a novel inhibitor of SIRT6, could inhibit angiogenesis both in vitro and in vivo. Molecular studies demonstrated that **8a** could downregulate the expression of angiogenesis-related proteins in HUVEC cells. Moreover, in pancreatic cancer, **8a** was able to downregulate the expression of CD31, a marker protein of angiogenesis, and inhibit angiogenesis. Therefore, **8a** could be used as a potential antiangiogenic and cancer therapeutic agent.

In the present study, compound **8a** showed significant inhibition of several angiogenic processes in HUVEC cells, including tube formation and migration, consistent with the clinical use of several antiangiogenic drugs such as monoclonal antibodies (bevacizumab and ramucirumab) [50,51]. Interestingly, **8a** induced almost no apoptosis in HUVEC cells in a short period at high doses (50 μM), indicating that **8a** has low cytotoxicity towards HUVEC cells and, therefore, the antiangiogenic effect of **8a** is not due to its cytotoxicity towards HUVEC cells.

We further investigated that **8a** could upregulate the acetylation level of histone H3, a downstream target protein of SIRT6, indicating that **8a** could inhibit the deacetylase activity of SIRT6 at the HUVEC cell level. VEGF is a key proangiogenic factor that plays an important role in angiogenesis and tumor metastasis [49,52]. It has been reported that SIRT6 promotes HUVEC cell migration, invasion, and angiogenesis under normoxic or hypoxic conditions by regulating HIF-1α and thereby promoting the expression of several angiogenic factors, including Ang1, Ang2, PDGF-BB, VEGF, and ET-1 [9]. Our results found that inhibitor **8a** of SIRT6 inhibits VEGF, p-VEGFR2, and HIF-1α expression and suppresses angiogenesis, consistent with the knockdown of SIRT6 in BMVEC and HUVEC cells [9,10]. N-cadherin is present in the adhesion complex between endothelial and pericytes, stabilizes cell–cell junctions, promotes cell migration, and is also involved in tumor progression and metastasis and is closely associated with the formation of blood vessels and the maintenance of vascular integrity [28,29,53]. We found that **8a** could inhibit the expression of N-cadherin. AKT and ERK pathways play an important role in the formation of blood vessels [32,33]. We also demonstrated that **8a** could inhibit the activation of AKT and ERK pathways. Thus, **8a** could exert an antiangiogenic effect by regulating AKT and ERK pathways and the expression of VEGF, N-cadherin, and HIF-1α. However, the explicit mechanism by which **8a** exerts its effect remains to be further explored.

Zebrafish embryos have become a very attractive model for studying vascular development due to their high genetic similarity to humans, external fertilization, rapid embryonic development, and high embryonic transparency [54,55,56]. Zebrafish embryos first develop to form the dorsal aorta and posterior main veins. Secondary vessels are then formed through sprouting angiogenesis, such as the intersegmental vessels [57]. We selected a transgenic zebrafish model *(Tg (flk1:EGFP))* to study the effect of **8a** on angiogenesis in vivo. We found that **8a** significantly inhibited the angiogenesis of intersegmental vessels in zebrafish, with no significant effect on mature vessels formed before **8a** treatment. This also suggests to us that **8a** may have a relatively small and safer effect on the blood vessels of normal organisms.

Pancreatic cancer is a highly aggressive malignancy with poor late-stage survival rates. The 5-year survival rate for patients with pancreatic cancer is the lowest, no more than 5%, and the median survival is only 6 months [58]. Angiogenesis is a key step in the growth and spread of malignant diseases, including pancreatic cancer [59]. These studies illustrate that targeting angiogenesis is an important strategy for tumor treatment. Previous reports have demonstrated that SIRT6 could promote the expression of inflammatory factor IL-8, which could promote local inflammation and further promote angiogenesis, playing a key role in the occurrence and metastasis of pancreatic cancer [12,13,14]. Thus, **8a**, an inhibitor of SIRT6, may inhibit angiogenesis in pancreatic cancer. In our study, we investigated the effect of **8a** on the angiogenesis of pancreatic cancer and found that **8a** could inhibit the expression of CD31 and HIF-1α, indicating that **8a** also has an inhibitory effect on angiogenesis in pancreatic cancer. Our previous study also demonstrated that **8a** inhibits the proliferation of pancreatic cancer and promotes the sensitivity of pancreatic cancer to gemcitabine in vivo and in vitro [21]. These results also suggest that the combination of **8a** with other drugs may be a promising antitumor strategy. For instance, the enzyme nicotinamide N-methyltransferase (NNMT) is overexpressed in pancreatic cancer, contributing to aggressiveness [60,61]. By methylating nicotinamide, NNMT can regulate the NAD levels reducing the amount of free nicotinamide, which could be converted into NAD through the NAD-salvage pathway, thus reducing the substrate for sirtuins activity [62]. In the light of these observations, coupling the antiangiogenic activity of **8a** with NNMT inhibitors [63,64,65] may be a promising strategy to improve the outcome of patients with pancreatic cancer, a malignancy that displays little therapeutic options. Taken together, these data suggest that **8a** has an antiangiogenic effect and is a potentially effective agent for cancer treatment.

## 4. Materials and Methods

### 4.1. Drugs and Reagents

Compound **8a** (purity > 95%) was synthesized as previously reported [21]. Compound **8a** was dissolved in double steaming water.

### 4.2. Cell Lines and Cell Culture

Human umbilical vein endothelial cells (HUVECs) were purchased from the Cell Resource Center of the Shanghai Institute for Biological Sciences, Chinese Academy of Sciences. HUVECs in passages 3 to 6 were used for experiments. HUVECs were cultured in Dulbecco’s modified Eagle’s medium (DMEM, GIBCO, Grand Island, NY, USA) supplemented with 10% (*v*/*v*) fetal bovine serum (FBS) (PAN-Biotech GmbH, Lot Number # ST200707), penicillin (50 units/mL), and streptomycin (50 μg/mL). Cells were cultured in a constant temperature incubator at 37 °C and 5% CO_2_ concentration.

### 4.3. CCK8 Assay

The effect of compound **8a** on the viability of HUVEC cells was determined by the Cell Counting Kit-8 (CCK8, Beyotime, Shanghai, China). HUVEC cells were seeded in 96-well plates (Corning, New York, NY, USA) at a density of 1 × 10^4^ cells/well. After incubation overnight, the HUVEC cells were incubated with different concentrations of **8a** (0, 3.125, 6.25, 12.5, 25, and 50 μM) for 24 h. The CCK8 solution was added to the well plates and incubated for 2–4 h in a 37 °C incubator. The absorbance was detected at 450 nm using a multifunctional enzyme marker (Bio-Tek, Winooski, VT, USA), and the cell viability (%) was calculated by the absorbance.

### 4.4. Cell Apoptosis Analysis

HUVEC cells were seeded in six-well culture plates at a density of 2 × 10^5^ cells/well. After incubation overnight, HUVEC cells were added to fresh DMEM medium with different concentrations of **8a** (0, 3.125, 6.25, and 12.5 μM) for 24 h. After the treatment, cells were harvested into centrifuge tubes and washed three times using precooled PBS buffer. Apoptotic cells were stained with Muse™ Annexin V &Dead Cell assay kit (Muse TM Cell Analyzer, Millipore (catalog no. MCH100105)) according to the manufacturer’s instruction. The cell samples were then analyzed by flow cytometry (Muse TM Cell Analyzer, Millipore, Bedford, MA, USA).

### 4.5. Scratch Wound-Healing Assay

A scratch wound-healing assay was used to detect cell migration. HUVEC cells were seeded in six-well culture plates at a density of 2 × 10^5^ cells/well. When the HUVEC cell confluence reached 80%, scratch wounds were made by a 100 μL pipette tip in a sterile environment. After washing three times with PBS buffer, HUVEC cells were added to a fresh DMEM medium containing 1% FBS with different concentrations of **8a** (0, 3.125, 6.25, and 12.5 μM) for 24 h. Images were recorded at 0 and 24 h by microscope (NIB-100, Novel Optics, Ningbo, China) after the cells were scratched. Each wound area was measured by using Image J software version 1.53 (Medical Cybernetics, New York, NY, USA), and the cell migration rate was assessed as follows: wound healing area (%) = (A_0 h_ /A_n h_)/A_0 h_ × 100, where A_0_ represents the original wound area (t = 0 h) and A_n_ represents the area of the wound at the time of detection (t = n h).

### 4.6. Transwell Migration Assay

HUVEC migration assays were evaluated in transwell plates (8 µm pore size, Corning) as previously described [66]. Briefly, HUVEC cells were trypsinized and then counted with a Muse cell analyzer (Millipore, USA). Cells were diluted by DMEM medium supplemented with 1% bovine serum albumin (BSA) and different concentrations of **8a** (0, 3.125, 6.25, and 12.5 μM) and seeded into the upper chamber at a density of 2 × 10^4^ cells/well. Then, the bottom chamber was added to DMEM medium containing 10% FBS as an attractant factor and different concentrations of **8a** (0, 3.125, 6.25, and 12.5 μM). After 24 h of incubation at 37 °C, the upper chambers were fixed with paraformaldehyde (4%) for 15 min, then washed three times with precooled PBS buffer and stained with crystal violet (0.1%) at room temperature for 15 min, the floating color washed off with PBS, and then the upper surface of the membrane cells was carefully removed by a cotton swab. Randomly selected fields of view were photographed using a microscope (NIB-100, CHN, original magnification). Then, the crystalline violet in the chambers was dissolved using acetic acid at a concentration of 33%, and the solution from each group was then added to a 96-well plate. The absorbance was detected at 570 nm using a multifunctional enzyme marker (Bio-Tek, USA).

### 4.7. In Vitro Tube Formation Assay

The tube formation assay on Matrigel (Corning (BD Biocoat), CA, USA) in vitro was used to assess the effect of compound **8a** on HUVEC tube formation capacity. In brief, the Matrigel was thawed overnight at 4 °C and added to 96-well plates with 60 μL per well. The plates were placed in a 37 °C incubator to solidify for 1 h. HUVEC cells were diluted by DMEM medium supplemented with 10% FBS and different concentrations of **8a** (0, 3.125, 6.25, and 12.5 μM), seeded into Matrigel at a density of 1 × 10^4^ cells/well, and incubated at 37 °C in an incubator for 6 h. Moreover, the tube formation was recorded by a light microscope (NIB-100, CHN, original magnification). The total length of the tubes was quantitatively evaluated by Image J software v1.53.

### 4.8. Zebrafish Embryo Assay

Angiogenesis experiments in vivo were performed using the Tg(flk1:EGFP) transgenic zebrafish line that can be directed to express an enhanced green fluorescent protein (EGFP) via endothelial-specific flk1 promoter, which, in turn, labels vascular endothelial cells [67,68]. Adult zebrafish were kept in an environment with a water temperature of about 28.5 °C, pH in the range of 6.8–7.2, 14 h:10 h of light: dark cycle, and fed with the appropriate amount of food on time. Before fertilization, male and female zebrafish were placed on both sides of the tank in a ratio of 1:2. When they finished free mating, their fertilized embryos were collected and washed with embryo culture water (5.4 mol/L KCl, 0.137 mol/L NaCl, 0.44 mol/L K_2_HPO_4_, 0.25 mol/L Na_2_HPO_4_, 1.0 mol/L MgSO_4_, 1.3 mol/L CaCl_2_, and 4.2 mol/L NaHCO_3_) and incubated in a constant temperature incubator at 28.5 °C. At about 20 hpf, the dorsal aorta (DA) and cardinal vein (CV) of zebrafish embryos were developed and new vessels started to extend towards their trunk and tail, and the intersegmental vessels were developed and blood circulation started to appear at about 48 hpf. To test the effect of compound **8a** on zebrafish angiogenesis, zebrafish embryos at 20 hpf were placed in 12-well plates with 25 embryos per well. The number of intersegmental vessels (ISVs) was counted after 48 h of administration of different concentrations of **8a** in the zebrafish culture medium. The ISVs formation was recorded by an inverted fluorescence microscope (DM6000, Leica, Wetzlar, Germany). The total length of ISVs was quantitatively evaluated by Image J software v1.53.

### 4.9. Western Blot Analysis

HUVEC cells were seeded in six-well culture plates at a density of 2 × 10^5^ cells/well. After incubation overnight, HUVEC cells were added to fresh DMEM medium with different concentrations of **8a** (0, 3.125, 6.25, and 12.5 μM) for 24 h. Cells were harvested, then washed twice with PBS buffer and lysed in a lysis solution (solarbio, R0010) containing protease inhibitor (TargetMol, Boston, MA, USA) and 1 mM PMSF. Total protein concentrations were assessed with BCA Protein Assay Kit (NCM Biotech, Suzhou, China). Then, protein samples were boiled in loading buffer for 10 min and stored at 20 °C. Equal amounts of protein samples (30 μg) were separated in SDS-PAGE at a separation gel concentration of 12.5%, and then the proteins were transferred to 0.45 μm PVDF membranes (Merck Millipore, Bedford, MA, USA). The PVDF membranes were blocked by 5% BSA in TBST buffer (10 mM Tris–HCl, 100 mM NaCl, 0.1% Tween-20, pH 7.5) at room temperature for 1 h. The blocked PVDF membranes were incubated with primary antibodies: β-Actin (1:2000, Cell Signaling Technology, Danvers, MA, USA, Cat#4970S), Acetyl-Histone H3 (Lys9) (1:2000, Cell Signaling Technology, Cat#9649), VEGF (1:1000, ZEN-BIOSCIENCE, R26073), N-Cadherin (1:1000, Cell Signaling Technology, Cat#13116), VEGFR2 (1:1000, Cell Signaling Technology, Cat#9698), p-VEGFR2 (1:1000, Cell Signaling Technology, Cat#3770), Histone H3 (1:2000, Cell Signaling Technology, Cat#9715S), AKT (1:1000, Cell Signaling Technology, Cat#9272S), Phospho-AKT (Ser473) (D9E) (1:1000, Cell Signaling Technology, Cat#4060S), ERK1/2 (1:1000, Cell Signaling Technology, Cat#4695), Phospho-p44/42 MAPK (ERK1/2) (Thr202/Tyr204) (1:1000, Cell Signaling Technology, Cat#4370S), and HIF-1α (1:1000, Cell Signaling Technology, Cat#36169) at 4 °C overnight. The PVDF membranes were washed three times by TBST for 10 min each time. Subsequently, the PVDF membranes were incubated with the respective horseradish peroxidase-conjugated secondary antibodies at room temperature for 1–2 h. The PVDF membranes were washed three times again by TBST for 10 min each time. Finally, the bands were detected by an electrochemiluminescence detection system (Tanon, Beijing, China) and were quantified using Image J software v1.53.

### 4.10. Immunohistochemical Staining

Female BALB/c nude mice (5 weeks old) were obtained from Beijing Vital River Laboratories (Beijing, China). All procedures were performed in adherence with international ethical guidelines and approved by the Animal Ethics Committee of the Ocean University of China. All mice were placed under standard conditions with free access to food and water. BXPC-3 cells (2 × 10^6^) were injected into the flank region of nude mouse. After the tumors’ volume had grown to 100 mm^3^, the mice were treated with **8a** (20 mg/kg) or vehicle every 2 days. Mice tumor tissues were removed. The tissue sections were dewaxed using xylene for 30 min and then rehydrated. The tissue sections were incubated with primary antibody of anti-CD31 (Cell Signaling Technology, Cat#77699) at 1:100 dilution at 4 °C overnight. Subsequently, the tissue sections were incubated with a universal secondary antibody (Maixin Biotechnology, Fuzhou, China, KIT-9707). The color development reaction was then performed using DAB solution and then counterstained with hematoxylin. Images were captured with inverted fluorescence microscope (DM6000, Leica, Wetzlar, Germany).

### 4.11. Statistical Analysis

All data are presented as the mean ± s.d. of at least three independent biological experiments (shown as error bars). Statistical differences were evaluated using one-way analysis of variance (ANOVA) or two-tailed Student’s *t*-tests. All statistical analyses were conducted using GraphPad Prism 8 software. *p* values less than 0.05 were considered statistically significant (*, *p* < 0.05; **, *p* < 0.01; ***, *p* <0.001).

## 5. Conclusions

In this study, we demonstrated for the first time that **8a**, a novel inhibitor of SIRT6, could inhibit HUVEC cell migration and tube formation. Molecular studies demonstrated that **8a** could inhibit angiogenesis by suppressing the activation of AKT and ERK pathways and the expression of VEGF, N-cadherin, p-VEGFR2, and HIF-1α in HUVEC cells. Moreover, **8a** significantly inhibited the angiogenesis of intersegmental vessels in zebrafish embryos. Importantly, in the pancreatic cancer xenograft mouse model, **8a** was able to downregulate the expression of CD31, a marker protein of angiogenesis, and inhibit angiogenesis. Therefore, these findings suggest that **8a** could be used as a potential antiangiogenic and cancer therapeutic agent.

## Data Availability

The data are contained within the article.
